# Mitochondrial aging and dysfunction in periodontitis

**DOI:** 10.1186/s12967-026-08766-y

**Published:** 2026-08-05

**Authors:** Yue Hu, Xuyan Li, Jianping Ge

**Affiliations:** 1 Department of Endodontics, School and Hospital of Stomatology, Shanghai Engineering Research Center of Tooth Restoration and Regeneration, Tongji University, Shanghai, 200072 P. R. China; 2 Department of Endodontics, Shanghai Engineering Research Center of Tooth Restoration and Regeneration, Shanghai Tongji Stomatological Hospital and Dental School, Tongji University, NO 399 Yanchang Zhong Road, Shanghai, P.R. China

**Keywords:** Periodontitis, Mitochondrial aging, Cellular senescence, Mitochondrial dysfunction

## Abstract

Periodontitis is a highly prevalent biofilm-associated chronic inflammatory disease that is a major cause of tooth loss and is linked to increased risk of cardiovascular disease, diabetes, adverse pregnancy outcomes, and other systemic disorders. Beyond classical concepts of plaque-induced inflammation, accumulating evidence suggests that mitochondrial dysfunction and mitochondrial aging are closely associated with the inflammatory and degenerative periodontal microenvironment. Hallmarks of mitochondrial aging, including excessive mitochondria-derived reactive oxygen species, mtDNA damage and leakage, impaired NAD⁺/sirtuin signaling, disturbed mitochondrial dynamics, defective mitophagy, and metabolic reprogramming, are observed in gingival epithelial cells and fibroblasts, periodontal ligament fibroblasts (PDLCs) and stem cells (PDLSCs), osteoblasts, osteoclasts, osteocytes, and resident and infiltrating immune cells. These changes are closely linked to cellular senescence, senescence-associated secretory phenotype (SASP), NLRP3 inflammasome activation, and uncoupling of bone formation and resorption. Extracellular vesicles can mediate the transfer of damaged mitochondria and regulatory nucleic acids between immune cells and mesenchymal stem cells. Through this process, they may reshape the stem cell niche, influence periodontal bone loss, and modulate orthodontic tooth movement. This review integrates current evidence on mitochondrial aging across periodontal cell populations and delineates a mitochondrial dysfunction–cellular senescence–SASP axis in periodontal tissue breakdown. It further summarizes mitochondria-targeted interventions, including MitoQ, resveratrol, coenzyme Q10, strategies that enhance mitophagy in PDLSCs, mitochondrial transplantation, and engineered exosomes, outlining a framework in which mitochondrial imbalance contributes to periodontal inflammation and tissue breakdown and may represent a potential therapeutic target.

## Introduction

Periodontitis is a chronic inflammatory disease initiated by dental plaque biofilms. It primarily affects the periodontal supporting tissues, including the gingiva, periodontal ligament, and alveolar bone, and it is now recognized as one of the most common oral health problems worldwide [1, 2]. Periodontitis often starts as gingivitis; without timely plaque control, inflammation can extend into deeper periodontal structures, eventually leading to tooth mobility and tooth loss [2, 3]. The number of people living with severe periodontitis increased steadily from 1990 to 2019 and surpassed one billion by 2019, with substantial impacts on chewing function and quality of life [1]. Large epidemiologic surveys suggest that about 40% of adults aged 30 years and older have periodontitis to some extent, and roughly 10% have severe disease [4, 5]. Mechanistically, periodontitis is thought to begin with a disruption of the microbial community in the gingival sulcus and periodontal pockets, which in turn triggers a dysregulated immune-inflammatory response and progressive tissue damage [6, 7].

Mitochondrial aging refers to a progressive decline in mitochondrial integrity and adaptive capacity that occurs with chronological aging and prolonged chronic stress. It is characterized by the accumulation of mtDNA damage, sustained mitochondrial ROS (mtROS) production, reduced NAD^+^/sirtuin signaling, impaired mitochondrial biogenesis, disrupted mitochondrial dynamics, and defective mitophagy [8, 9]. These age-associated mitochondrial alterations can reduce oxidative phosphorylation efficiency, weaken mitochondrial quality control, and promote a persistent low-grade inflammatory state known as inflammaging [10–15]. In periodontitis, gingival fibroblasts from affected patients show excessive mitochondrial ROS production and abnormal opening of the mitochondrial permeability transition pore, which can lead to mitochondrial DNA leakage and loss of membrane potential [16]. These mitochondrial disturbances may amplify downstream inflammatory signaling and thereby contribute to periodontal tissue destruction [17, 18]. In addition, dysregulated mitophagy may prevent damaged mitochondria from being cleared efficiently, thereby sustaining local inflammation once mitochondrial damage has occurred [19–21]. These findings indicate an association between periodontitis, aging, and mitochondrial dysfunction [18, 22–24].

This review examines hallmark features of mitochondrial aging in periodontitis, including increased mtROS, cytosolic leakage of mtDNA, and reduced NAD⁺ levels. We also summarize mitochondrial dysfunction in major periodontal cell types, including gingival epithelial cells, fibroblasts, PDLCs, PDLSCs, osteoblasts, osteoclasts, and osteocytes. We then discuss how these changes may contribute to disease progression. Finally, we discuss emerging therapeutic approaches such as mitochondria-targeted small molecules, mitochondrial transplantation, and engineered exosomes, and consider their potential roles in treating periodontitis, with the aim of informing future clinical strategies that target mitochondria.

## Mitochondrial aging as a central feature of periodontal disease

### Mitochondrial aging and stress-induced mitochondrial dysfunction in periodontal disease

Aging is accompanied by progressive mitochondrial dysfunction, but mitochondrial dysfunction is not specific to aging and can also be induced by inflammatory, metabolic, or microbial stress [11]. In aged organisms, the electron transport chain, particularly complexes I and III, exhibits increased electron leakage, which leads to a sustained elevation in mtROS [25].

**Fig. 1:**
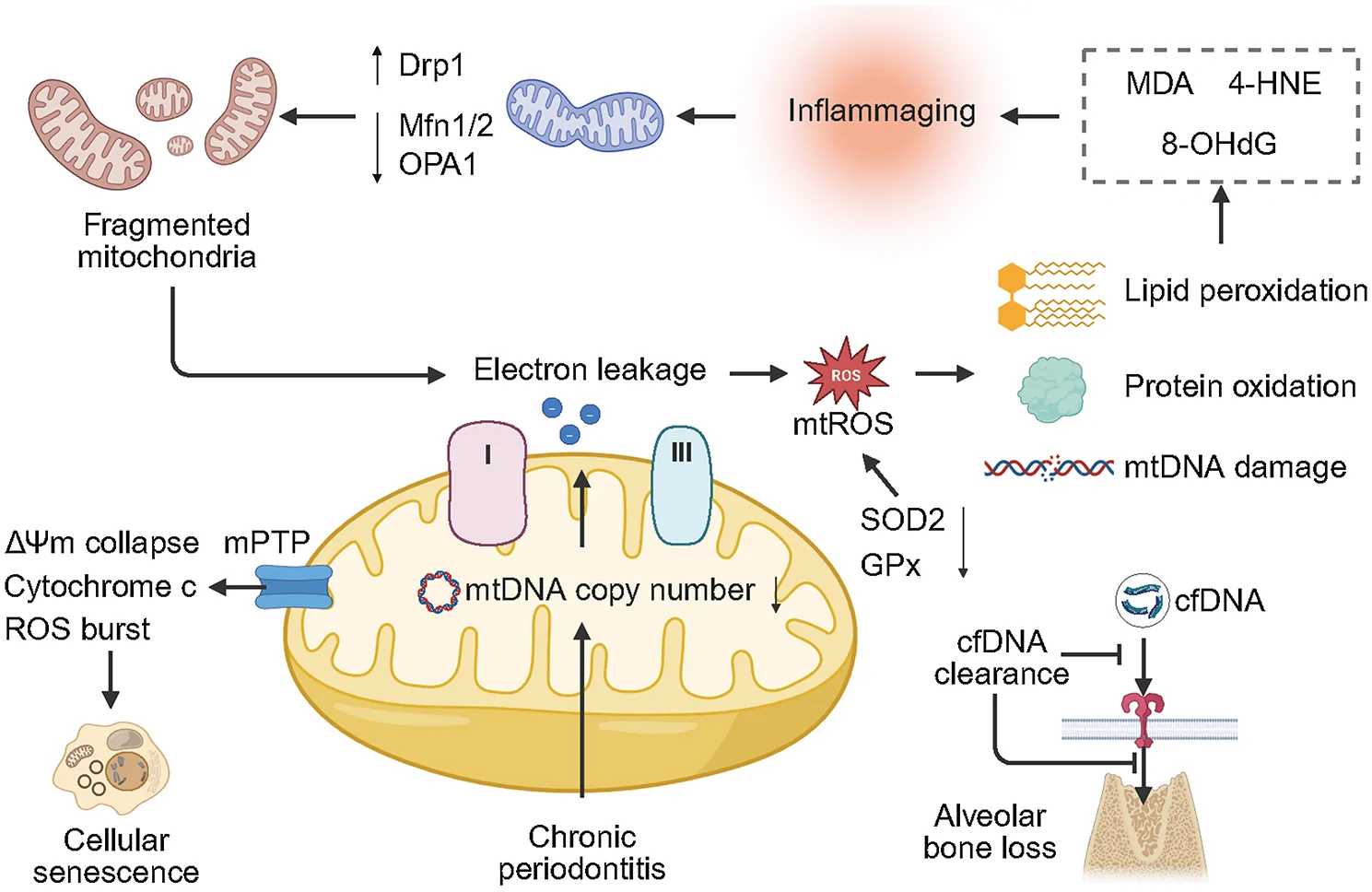
Mitochondria-derived ROS drive inflammaging in senescent and periodontitis cells. In aged organisms, electron leakage from mitochondrial electron transport chain complexes I and III causes a persistent increase in mitochondria-derived mtROS. mtROS induce lipid peroxidation, protein oxidation, and oxidative damage to mtDNA, generating damage markers such as malondialdehyde, 4-hydroxynonenal, protein carbonyls, and 8-hydroxy-2’-deoxyguanosine, thereby driving inflammaging. In senescent cells, activation of the fission factor Drp1 together with downregulation of the fusion proteins Mfn1/2 and OPA1 results in a fragmented mitochondrial network that is more prone to electron leakage. Moreover, the expression or activity of mitochondrial antioxidant enzymes such as SOD2 and glutathione peroxidase is reduced, weakening the clearance of superoxide anion and hydrogen peroxide and further amplifying mtROS accumulation. Under sustained stress, abnormal opening of the mPTP triggers mitochondrial membrane potential collapse, cytochrome c release, and additional ROS bursts, thereby accelerating cellular senescence. Cells derived from chronic periodontitis lesions continuously exhibit higher mtROS levels and lower mtDNA copy numbers, which disrupt the integrity of the electron transport chain, reduce oxidative phosphorylation efficiency, and further promote excessive mtROS generation through electron leakage, establishing a vicious cycle in which damage and stress amplify each other. Removal of local cell-free DNA significantly decreases TLR9-dependent proinflammatory cytokine levels and attenuates alveolar bone loss. mtROS, mitochondria-derived reactive oxygen species; mtDNA, mitochondrial DNA; Drp1, dynamin-related protein 1; Mfn1/2, mitofusin 1/2; OPA1, optic atrophy 1; SOD2, superoxide dismutase 2; mPTP, mitochondrial permeability transition pore; ROS, reactive oxygen species; TLR9, Toll-like receptor 9

In patients with chronic periodontitis, gingival tissues and periodontal ligament cells commonly display disrupted mitochondrial morphology, impaired respiratory function, and elevated levels of ROS (Fig. 1) [26]. Under oxidative stress conditions, human PDLCs show fragmented cristae, decreased mitochondrial membrane potential, and reduced ATP production, accompanied by a marked increase in mtROS [27]. Upon stimulation with periodontitis-associated pathogens, such as *Porphyromonas gingivalis* (*P. gingivalis*), or its lipopolysaccharide, mitochondrial pathways in gingival fibroblasts are activated. ROS promote abnormal opening of the mPTP, which induces mtDNA extrusion. Persistent mtDNA release can function as a damage-associated molecular pattern (DAMP), helping to perpetuate inflammation [17]. These findings indicate that periodontal pathogens can induce mitochondrial dysfunction and DAMP signaling, but they do not establish mitochondrial dysfunction as an initiating event in periodontitis. In addition, recent reviews report elevated mtROS in gingival epithelial cells, PDLSCs, osteoblasts, and infiltrating immune cells in the periodontitis microenvironment [26]. These findings mainly reflect oxidative stress-, pathogen-, or inflammation-induced mitochondrial dysfunction rather than mitochondrial aging itself. Although they share features of mitochondrial aging, such as mtROS accumulation, mPTP opening, and mtDNA release, they do not constitute direct evidence of mitochondrial aging. However, they may contribute to or accelerate age-related mitochondrial decline.

Excess mtROS impairs oxidative phosphorylation (OXPHOS). It is often accompanied by reduced mitochondrial membrane potential, decreased ATP production, and lower respiratory chain activity, indicating compromised cellular bioenergetic capacity [11]. At the same time, mtROS induce oxidative injury to multiple macromolecules by promoting lipid peroxidation, protein oxidation, and DNA damage, with mtDNA being particularly susceptible. Commonly used indicators of this damage include malondialdehyde (MDA), 4-hydroxy-2-nonenal (4-HNE), protein carbonyls, and 8-hydroxy-2’-deoxyguanosine (8-OHdG) [28]. Because many of these lesions are difficult to reverse or inefficiently repaired, they can accumulate over time, erode tissue function, and contribute to inflammaging [29]. The age-associated increase in mtROS is not only a consequence of reduced OXPHOS performance. It also reflects broader defects in mitochondrial quality control, including altered fusion–fission dynamics, impaired mitophagy, and reduced mitochondrial biogenesis [11]. In senescent cells, activation of the fission protein dynamin-related protein 1 (Drp1) and downregulation of the fusion proteins mitofusin 1 (Mfn1), mitofusin 2 (Mfn2), and optic atrophy 1 (OPA1) promote a fragmented mitochondrial network that is more prone to electron leakage and functional disruption [30]. At the same time, the expression or activity of mitochondrial antioxidant enzymes such as superoxide dismutase 2 (SOD2) and glutathione peroxidase decreases, which weakens the clearance of superoxide and hydrogen peroxide and further magnifies mtROS accumulation [28]. Under conditions of persistent stress or calcium overload, abnormal opening of the mPTP leads to collapse of the membrane potential, release of cytochrome c, and additional bursts of ROS, thereby accelerating cellular senescence [31].

MtDNA is a circular genome located within mitochondria. Because it lacks histone protection and has relatively limited repair capacity, mtDNA is particularly vulnerable to base oxidation and double strand breaks in an oxidative stress environment associated with aging [32]. With advancing age and increasing chronic inflammatory burden, point mutations, large scale deletions, and replication errors gradually accumulate in mtDNA. When they accumulate over time or occur in aged tissues, these alterations are considered molecular features of mitochondrial aging [32]. Studies have shown that gingival tissues from patients with chronic periodontitis frequently display a significant increase in a large mtDNA deletion of approximately 5000 base pairs. This rise is accompanied by higher levels of oxidative damage markers, including 8-OHdG, which supports the presence of early mitochondrial genome injury in the local periodontal milieu [33]. Whole mitochondrial genome sequencing has also identified multiple somatic mtDNA mutations in gingival tissues from patients with chronic periodontitis. Importantly, these mutations are observed in periodontal samples but not in peripheral blood, suggesting that the oxidative conditions within periodontal lesions favor tissue specific accumulation of mtDNA heterogeneity [34]. In patients who have chronic periodontitis together with coronary heart disease, certain somatic mtDNA mutations have been reported in both periodontal tissues and coronary artery specimens. This overlap raises the possibility that periodontium related mtDNA damage contributes to biological processes shared by oral disease and cardiovascular comorbidity [35]. In vitro, gingival fibroblasts derived from chronic periodontitis lesions maintain higher mtROS levels and show reduced mtDNA copy number over time [17]. These findings suggest that mtDNA abnormalities may disrupt the integrity of the electron transport chain and further promote mtROS overproduction through increased electron leakage, forming a vicious cycle in which damage and stress amplify each other [36].

Clinical studies report higher concentrations of cell free DNA in gingival crevicular fluid and saliva in patients with periodontitis compared with healthy controls. In these studies, cell free DNA levels also tend to increase with clinical measures of disease severity, including probing depth and bleeding indices [37, 38]. Cell free DNA in these fluids reflects a mixture of microbial and host nucleic acids, and can include host derived mtDNA fragments in addition to bacterial DNA [39]. In ligature-induced periodontitis mouse, local removal of extracellular DNA using cationic surface nanoparticles has been shown to reduce toll-like receptor (TLR) 9 dependent proinflammatory cytokine production and to lessen alveolar bone loss. These results provide functional evidence that cell free DNA, particularly CpG rich mtDNA, plays a pathogenic role in periodontal bone destruction [39]. Cellular study further show that gingival fibroblasts derived from chronic periodontitis patients maintain a high level of mtDNA release even after continuous passaging in vitro, with significantly increased mtDNA detectable in both the cytosol and culture supernatant [17]. This release is closely associated with intracellular ROS accumulation and persistent opening of the mPTP [17]. The same study reported that *P. gingivalis* lipopolysaccharide can induce a similar mtDNA leakage phenotype in gingival fibroblasts from healthy donors. This finding suggests that periodontal pathogens can enhance DAMP signaling by increasing ROS production, altering mitochondrial membrane permeability, and triggering mtDNA release. Even after removal of the stimulus, this abnormal mtDNA extrusion can persist as a form of cellular memory that maintains a proinflammatory, periodontitis prone state [17].

### The NAD⁺/sirtuin signaling axis linking mitochondrial stress and periodontal inflammation

During organismal aging, NAD⁺ levels gradually decline in many tissues. This reduction directly weakens the deacetylase activity of the Sirtuin family, particularly sirtuin (SIRT) 1 and SIRT3, and promotes the onset and progression of aging related chronic low-grade inflammation [40]. The decrease in NAD⁺ results from persistent activation of NAD⁺ consuming enzymes such as CD38 and poly ADP ribose polymerases, as well as from reduced efficiency of NAD⁺ salvage pathways [41]. Together, these changes suppress mitochondrial biogenesis and mitophagy and diminish the clearance capacity for mtROS, which makes senescent cells more prone to accumulate oxidative damage. SIRT3 is localized in the mitochondrial matrix. It regulates the acetylation of key enzymes involved in the tricarboxylic acid cycle, fatty acid oxidation, and oxidative phosphorylation [14]. When NAD⁺ levels decline, the deacetylase activity of SIRT3 is limited, which lowers the efficiency of the electron transport chain, sustains mtROS elevation, and ultimately aggravates damage to mtDNA and proteins [40].

SIRT1 is broadly expressed in periodontal tissues, and its activity is highly dependent on the availability of NAD⁺. A decrease in NAD⁺ therefore amplifies the inflammatory responses of periodontal tissues to bacteria and endotoxins [40, 42]. In human PDLCs, lipopolysaccharide stimulation markedly upregulates TLR4 and its downstream c-Jun N-terminal kinase (JNK) and NF-kappa B signaling pathways, and enhances the secretion of IL-1α, IL-6, IL-8, and TNF-α [43]. Overexpression of SIRT1 downregulates TLR4 and inhibits the JNK and NF-kappa B pathways, which reduces apoptosis and lowers the release of inflammatory cytokines [42, 44]. These findings confirm that SIRT1 attenuates periodontitis related inflammation by restraining innate immune receptor signaling [42]. Moreover, studies have shown that in the setting of chronic periodontitis, exosomes derived from PDLSCs regulate the Th17 and Treg balance by altering the expression profiles of miR-155-5p and SIRT1 [45]. Downregulation of miR-155-5p together with upregulation of exosomal SIRT1 contributes to suppression of proinflammatory Th17 responses and restoration of immune homeostasis [45]. These observations suggest that the NAD⁺/SIRT1 axis not only controls inflammatory programs in resident periodontal cells but also indirectly shapes the local adaptive immune microenvironment in the periodontium through exosome mediated intercellular communication [45]. In models of diabetic periodontitis, exposure of hPDLSCs to high glucose together with lipopolysaccharide reduces SIRT3 expression, accelerates cellular senescence, and impairs osteogenic differentiation [46]. By contrast, aged SIRT3-knockout mice provide more direct evidence linking impaired mitochondrial deacetylation to age-related periodontal breakdown. In this model, alveolar bone volume is reduced, osteoclast numbers are increased, and mitochondrial protein acetylation and CypD expression are elevated [46, 47]. When SIRT3 is downregulated, LRPPRC becomes more acetylated, oxidative phosphorylation is compromised, and mtROS levels rise. Over time, these mitochondrial defects are associated with PDLSC senescence and loss of function [14, 46]. In aged SIRT3-knockout mice, there is a pronounced loss of alveolar bone volume, an increased distance from the cementoenamel junction to the alveolar bone crest, and a higher number of osteoclasts. Together, these findings support the interpretation that SIRT3 deficiency worsens age related periodontal breakdown by favoring bone resorption and disrupting normal bone remodeling dynamics [14]. In the same model, investigators observed increased mitochondrial protein acetylation and elevated CypD expression in alveolar bone. These findings indicate that SIRT3 insufficiency may enhance mPTP signaling, increase mitochondrial vulnerability, and promote a higher propensity for cell death, thereby establishing a mechanistic link between structural damage and alveolar bone resorption [14]. In animal models of diabetic periodontitis, pharmacologic activation of SIRT3 improves mitochondrial respiratory function in PDLSCs, reduces oxidative stress and cellular senescence, and promotes alveolar bone regeneration. These results support restoration of the NAD⁺/SIRT3 axis as a potential strategy to reverse diabetes associated degenerative changes in periodontal tissues [46].

## Cell type specific mitochondrial defects in periodontal supporting tissues

### Gingival epithelial cells and fibroblasts as mitochondrial sentinels of the barrier

In healthy gingival epithelial cells and fibroblasts, mitochondria not only generate ATP through oxidative phosphorylation but also participate in the regulation of intracellular calcium homeostasis and apoptosis [48–50]. In addition, they function as key platforms for sensing danger signals within the innate immune system and are therefore essential for maintaining the structural and functional integrity of the gingival barrier [51, 52]. In gingival tissues and primary human gingival fibroblasts from patients with chronic periodontitis, characteristic ultrastructural abnormalities of mitochondria are frequently observed, including mitochondrial swelling, disrupted cristae, and electron lucent matrices. These changes are accompanied by reduced mitochondrial membrane potential and disturbed oxygen consumption profiles, indicating impaired mitochondrial function [17]. MtDNA copy number is decreased and mtDNA is slowly released into the cytosol and extracellular milieu [53]. These findings suggest compromised mitochondrial genome stability and imply that mtDNA may act as a damage associated molecular pattern that amplifies local inflammatory responses [17, 33]. Functional studies in human gingival fibroblasts have shown that cells derived from chronic periodontitis lesions exhibit higher levels of mitochondria derived ROS, lower mitochondrial membrane potential, and imbalanced respiratory chain activity even under basal conditions. These alterations are accompanied by increased secretion of inflammatory cytokines such as IL 6, IL 1β, and TNF α, which underscores the tight link between mitochondrial metabolic abnormalities and the inflammatory phenotype [48, 54]. Real time imaging further demonstrates that in fibroblasts from periodontitis patients, mtDNA continuously extrudes from swollen mitochondria into the cytosol and the extracellular space [55]. This phenomenon persists even after removal of lipopolysaccharide stimulation and following multiple passages in culture, indicating a form of cellular memory associated with persistent mitochondrial damage and aberrant mtDNA release [17]. At the mechanistic level, lipopolysaccharide derived from *P. gingivalis* can suppress the expression of pyruvate dehydrogenase kinase 2, promote opening of the mPTP, enhance mitochondrial ROS generation, and trigger large scale mtDNA leakage [56]. As a result, molecules that are normally confined within mitochondria are converted into danger signals that activate innate immune pathways [17].

### Metabolic reprogramming and mitochondrial failure in PDLCs and stem cells

PDLCs and PDLSCs are rich in mitochondria and rely primarily on oxidative phosphorylation to meet their energy demands, which is critical for maintaining periodontal tissue homeostasis and responding to mechanical loading [57]. Integrity of mitochondrial structure and function is therefore a central prerequisite for preserving the normal biological functions of these cells [26, 58]. In inflammatory and diabetes-associated models of periodontitis, PDLCs and PDLSCs commonly display mitochondrial damage, including increased mitochondrial volume, loosened matrix density, disrupted cristae, and vacuole-like degeneration, Such mitochondrial abnormalities are accompanied by impaired cellular metabolism and function, indicating that inflammatory and metabolic stress can profoundly disrupt mitochondrial homeostasis in periodontal cells [26, 59]. Seahorse based metabolic assays further show a decline in oxygen consumption rate, indicating impaired oxidative phosphorylation that cannot adequately support the high energy requirements of osteogenic differentiation and extracellular matrix synthesis [60]. In a high glucose environment, PDLSCs exhibit increased NADPH dependent ROS production, enhanced lactate generation, and elevated extracellular acidification rate, which indicate a shift from mitochondria centered oxidative phosphorylation to a glycolysis dominated metabolic state [60, 61]. Within this metabolic context, enhanced NADPH oxidase activity together with blockade of electron transport chain function leads to excessive intracellular ROS accumulation. Antioxidant defense systems such as glutathione and superoxide dismutase are continuously depleted, creating a persistent oxidative stress milieu [60]. Under high glucose conditions, the expression of annexin A2 (ANXA2) is downregulated in PDLCs and this change is accompanied by elevated ROS levels and mitochondrial dysfunction. These alterations further weaken osteogenic gene expression and the cellular capacity to resist damage, suggesting a close relationship between structural protein changes and mitochondrial homeostasis imbalance [62]. In vitro experiments have shown that combined high glucose and TNF α stimulation significantly increases ROS production and caspase 3 activation in PDLSCs, which aggravates proliferation arrest and apoptosis. Treatment with the antioxidant vitamin C can partially reverse these detrimental effects, further confirming that oxidative stress and mitochondrial injury are key mechanisms underlying cell dysfunction in an inflammatory and hyperglycemic environment [63–65]. Periodontitis and diabetic periodontitis jointly promote a metabolic switch in PDLCs and PDLSCs from a predominantly mitochondrial oxidative phosphorylation based aerobic metabolism to a state characterized by high flux aerobic glycolysis [26, 66, 67]. This reprogramming not only decreases the amount of ATP produced per unit of substrate but also increases the burden of lactate and ROS accumulation, creating a vicious cycle in which energy insufficiency and local inflammatory responses mutually aggravate each other [26]. In oxidative stress driven models of periodontitis, activation of the PTEN-induced kinase 1 (PINK1)/Parkin pathway to enhance mitophagy can partially reduce mitochondrial ROS load and improve periodontal bone remodeling [68]. These findings indirectly support the critical role of impaired mitochondrial quality control in PDLCs during disease progression [69, 70]. The metabolic and quality control abnormalities described above are often accompanied by decreased expression of mitochondrial deacetylases such as SIRT3 and altered levels of metabolic regulators including spermidine. Together, these changes weaken the ability of PDLSCs to clear damaged mitochondria and buffer oxidative stress, and provide a theoretical rationale for developing mitochondria targeted strategies to improve cell function [26].

### Mitochondrial control of osteoblast, osteoclast, and osteocyte function in the alveolar bone

The alveolar bone is a mineralized tissue that undergoes continuous remodeling through the coordinated actions of osteoblasts, osteoclasts, and osteocytes [71, 72]. In these cells, mitochondria are central organelles that support oxidative phosphorylation and ATP production, regulate ROS generation, and participate in intracellular signal transduction. These functions are essential for maintaining local bone homeostasis [73].

In elderly individuals and in bone loss models related to periodontitis, osteoblast lineage cells frequently display mitochondrial ultrastructural abnormalities, including swollen mitochondria, disrupted cristae, and reduced matrix electron density [74, 75]. These structural changes are accompanied by reduced mitochondrial membrane potential, diminished activity of respiratory chain complexes, and restricted ATP synthesis, which together lead to a marked decline in matrix synthesis and mineralization capacity [76]. The structural and bioenergetic defects coincide with sustained increases in mitochondria derived superoxide and hydrogen peroxide [77, 78]. Because antioxidant defenses such as SOD2 are insufficient, osteoblasts and osteocytes remain in a chronic oxidative stress state and are more susceptible to DNA damage, opening of the mPTP, and apoptosis, which ultimately compromises their osteogenic activity [73, 79].

In individuals with type 2 diabetes, elevated free fatty acid load and high glucose levels enhance mitochondrial fatty acid oxidation and electron leakage from the electron transport chain in osteoblasts [80]. Through the FFA-ROS-p53 pathway, these changes induce mitochondria dependent apoptosis and reduce the differentiation of bone marrow mesenchymal stem cells toward the osteogenic lineage. As a result, both the number and function of osteoblasts decline, which contributes to systemic and maxillofacial osteopenia [80]. It is reported that osteoblasts and supporting cells in the alveolar bone region exhibit decreased mitochondrial number, fragmented mitochondrial morphology, reduced membrane potential, and lower mtDNA copy number. These alterations are accompanied by impaired oxidative phosphorylation efficiency and lactate accumulation, providing a metabolic basis for diminished local bone regeneration and progressive alveolar bone loss [81].

Osteoclasts are inherently rich in mitochondria. Their differentiation and bone resorptive activity are highly dependent on ATP supply and require an appropriate level of mitochondria derived ROS as an amplification mechanism for receptor activator of nuclear factor kappa-B ligand (RANKL) signaling [56, 82]. In the inflammatory microenvironment of the alveolar bone, persistent RANKL stimulation together with reduced intracellular antioxidant capacity leads to excessive mtROS accumulation, which further enhances osteoclast formation, adhesion, and acid mediated demineralization [73]. In animal models of periodontitis and diabetic periodontitis, alveolar bone tissues show upregulated RANKL expression and relatively reduced osteoprotegerin (OPG) levels [83]. The resulting increase in the RANKL/OPG ratio positively correlates with mitochondrial dysfunction and ROS accumulation in osteoclasts, indicating that mitochondrial imbalance shifts the bone immune axis toward enhanced bone resorption and aggravates local trabecular thinning and cortical porosity [26].

Osteocytes are deeply embedded within the alveolar bone matrix and form a dense canalicular network through their long cellular processes [84]. Mitochondria are distributed along the cell bodies and processes to sustain local ATP supply, calcium homeostasis, and mechanosensory function [85]. When SOD2 deficiency or other factors lead to accumulation of mitochondrial superoxide in osteocytes, the canalicular structures become prone to disruption and collapse, and osteocyte survival decreases [77]. These changes impair the ability of osteocytes to perceive mechanical loading and microdamage and to coordinate appropriate remodeling responses [76]. In addition, mitochondrial oxidative stress promotes increased secretion of pro resorptive and matrix degrading factors by osteocytes, including RANKL, sclerostin, IL 6, and multiple matrix metalloproteinases [86]. This secretory profile closely mirrors the amplified local inflammation, uncoupled bone remodeling, and trabecular rarefaction observed in the alveolar bone of periodontitis patients and can be regarded as a functional manifestation of mitochondrial dysfunction in osteocytes [26].

## Mitochondrial dysfunction, cellular senescence, and SASP in periodontal breakdown

### Mitochondrial dysfunction-associated senescence and SASP amplification in periodontal cells

**Fig. 2:**
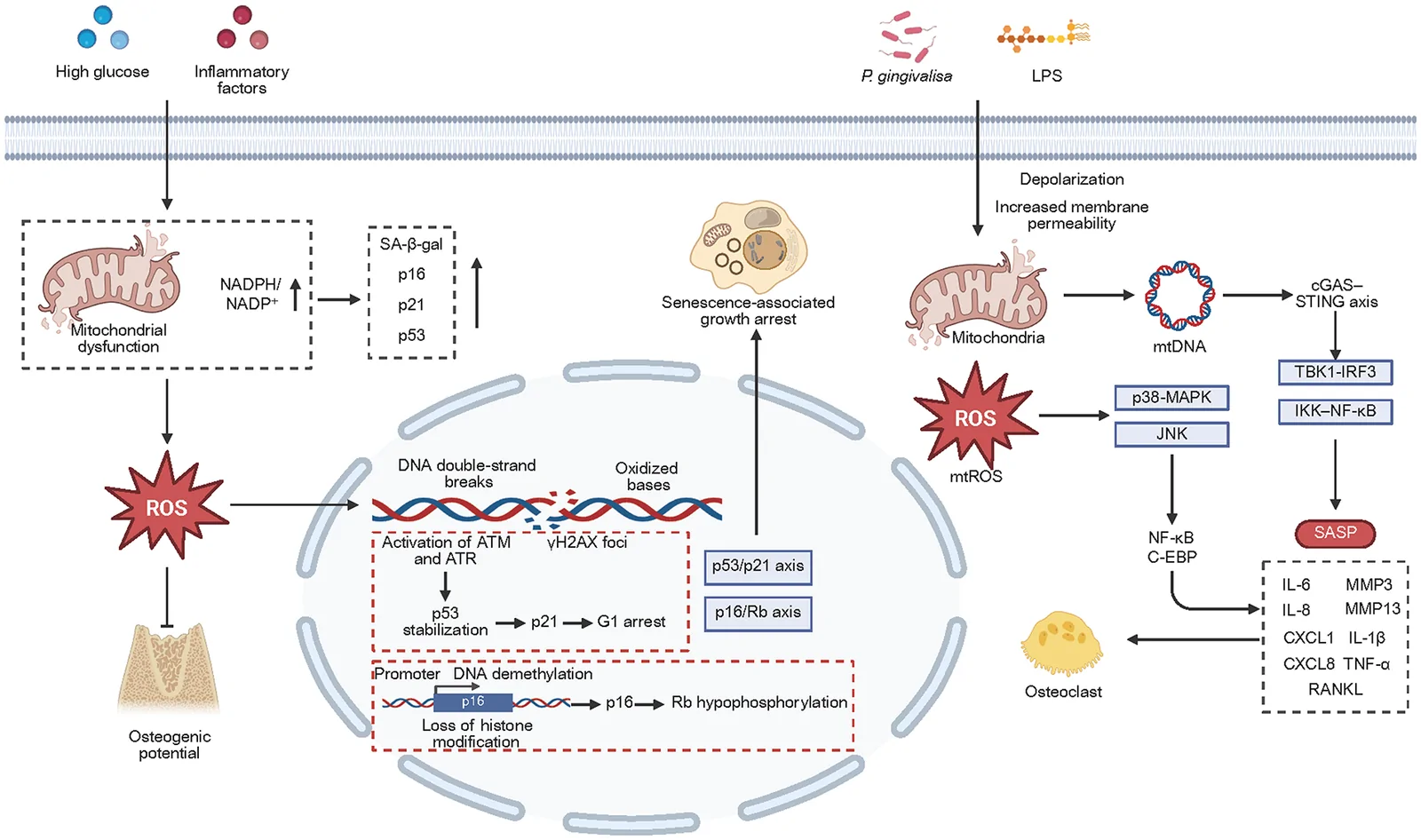
Mitochondria-driven senescence and SASP in periodontal cells. Mitochondrial dysfunction induces nuclear DNA double-strand breaks and oxidative base lesions through excessive production of ROS, thereby activating ATM and ATR and generating γH2AX foci. This stabilizes p53, upregulates p21, and arrests cells in the G1 phase. Concomitantly, DNA demethylation and loss of histone marks occur in the p16 promoter region, leading to sustained p16 overexpression and persistent hypophosphorylation of Rb. The p16/Rb axis, together with the p53/p21 axis, maintains cells in an irreversible senescence-associated growth arrest. Under high-glucose conditions combined with inflammatory cytokine stimulation in vitro, human PDLSCs display pronounced oxidative stress and mitochondrial dysfunction, along with increased SA-β-gal positivity and upregulation of the senescence-associated proteins p16, p21, and p53. In addition, high glucose markedly elevates intracellular NADPH levels and the NADPH/NADP⁺ ratio in PDLSCs, resulting in excessive ROS accumulation; inhibition of NADPH production or reduction of ROS levels can partially restore the osteogenic capacity of PDLSCs. Mitochondrial depolarization and increased membrane permeability promote leakage of a fraction of mtDNA into the cytosol. This cytosolic mtDNA is sensed as a DNA signal by cGAS, which then activates the TBK1–IRF3 and IKK–NF-κB pathways via the cGAS–STING axis, thereby inducing expression of inflammatory cytokines and chemokines and establishing a SASP network centered on IL-6, IL-8, CXCL1, and CXCL8. mtROS can activate the p38-MAPK and JNK pathways; activated p38 stabilizes the activity of NF-κB and the C-EBP family, causing senescent cells to continuously secrete large amounts of IL-6, IL-8, MMP3, and multiple CXCL chemokines. In human gingival fibroblasts exposed to *P. gingivalis* or its lipopolysaccharide, mitochondrial depolarization, elevated mtROS levels, and release of mtDNA from mitochondria into the cytosol and extracellular space can be observed. In alveolar bone osteocytes and osteoblastic lineage cells, LPS stimulation or senescence-associated stress can induce a decrease in mitochondrial membrane potential, a reduction in oxygen consumption rate, and an increase in ROS, accompanied by p21 upregulation and an increased proportion of SA-β-gal-positive cells; these senescent cells secrete high levels of IL-6, IL-1β, TNF-α, MMP13, and RANKL, thereby promoting osteoclast formation and accelerating bone loss. ROS, reactive oxygen species; ATM, ataxia telangiectasia mutated; ATR, ataxia telangiectasia and Rad3-related; γH2AX, phosphorylated H2AX (gamma-H2AX); p53, tumor protein p53; p21, cyclin-dependent kinase inhibitor 1 A; G1, G1 phase of the cell cycle; p16, cyclin-dependent kinase inhibitor 2 A; Rb, retinoblastoma protein; PDLSCs, periodontal ligament stem cells; SA-β-gal, senescence-associated β-galactosidase; NADPH, nicotinamide adenine dinucleotide phosphate (reduced form); NADP⁺, nicotinamide adenine dinucleotide phosphate (oxidized form); mtDNA, mitochondrial DNA; cGAS, cyclic GMP–AMP synthase; STING, stimulator of interferon genes; TBK1, TANK-binding kinase 1; IRF3, interferon regulatory factor 3; IKK, IκB kinase; NF-κB, nuclear factor kappa-B; IL-6, interleukin-6; IL-8, interleukin-8; CXCL1, C-X-C motif chemokine ligand 1; CXCL8, C-X-C motif chemokine ligand 8; SASP, senescence-associated secretory phenotype; mtROS, mitochondria-derived reactive oxygen species; p38-MAPK, p38 mitogen-activated protein kinase; JNK, c-Jun N-terminal kinase; C-EBP, CCAAT/enhancer-binding protein; MMP3, matrix metalloproteinase 3; *P. gingivalis*, Porphyromonas gingivalis; LPS, lipopolysaccharide; IL-1β, interleukin-1 beta; TNF-α, tumor necrosis factor-α; MMP13, matrix metalloproteinase 13; RANKL, receptor activator of nuclear factor κB ligand

Mitochondrial dysfunction is recognized as an important contributor to cellular senescence, particularly under persistent inflammatory, metabolic, or oxidative stress, and is characterized by reduced respiratory capacity in individual mitochondria, decreased membrane potential, and increased ROS production (Fig. 2) [87]. In DNA damage response (DDR)-type senescence pathways, mitochondrial dysfunction increases ROS production. Excessive ROS induces nuclear DNA double-strand breaks and oxidative base lesions [88]. These changes activate ATM/ATR signaling, increase γH2AX foci formation, stabilize p53, and upregulate p21, leading to G1-phase cell-cycle arrest. At the same time, in a background of chronic DNA damage and inflammatory stress maintained by mtROS, the p16 promoter undergoes DNA demethylation and loss of repressive histone marks, leading to persistent p16 overexpression and sustained hypophosphorylation of Rb. The p16/Rb axis together with the p53/p21 axis keeps cells not merely in a reversible quiescent state but locked in an irreversible senescent growth arrest [87, 88].

A related stress-induced senescence phenotype has been observed in periodontal ligament cells. In vitro exposure of human PDLSCs to high glucose together with inflammatory cytokines induces oxidative stress and mitochondrial dysfunction, accompanied by increased SA-β-gal positivity and upregulation of p16, p21, and p53. These findings indicate that metabolic and inflammatory stress can activate p53/p21- and p16/Rb-related senescence programs and reproduce several aging-like mitochondrial features in PDLSCs [60]. Metabolic analyses further show that high glucose significantly elevates NADPH levels and the NADPH to NADP⁺ ratio in PDLSCs. This metabolic shift enhances NADPH oxidase activity and promotes electron leakage from the mitochondrial respiratory chain, leading to excessive ROS accumulation. Inhibition of NADPH production or reduction of ROS levels partially restores the osteogenic capacity of PDLSCs, indicating that metabolic reprogramming and mitochondrial redox imbalance are key prerequisites that drive PDLSCs from functional impairment into a senescent program [60].

In addition to driving cell cycle arrest through DDR, mitochondrial damage connects directly to senescence-associated secretory phenotype (SASP) activation via mtROS and mtDNA release. In multiple cell types, mitochondrial depolarization and increased membrane permeability allow a fraction of mtDNA to leak into the cytosol. This cytosolic mtDNA is sensed as a DNA danger signal by cGAS and activates the cGAS-STING axis, which in turn stimulates TBK1/IRF3 and IKK/NF-κB pathways. These signaling cascades induce the expression of numerous inflammatory cytokines and chemokines and establish a SASP network centered on IL-6, IL-8, CXCL1, and CXCL8 [87, 88]. mtROS themselves also function as important redox signals. They can oxidize upstream sensor molecules and activate the p38 MAPK and JNK pathways. p38 has been identified as a critical signaling node for maintaining the SASP transcriptional program. Sustained p38 activation stabilizes NF-κB and C/EBP family activity. It also promotes the secretion of IL-6, IL-8, MMP-3, and CXCL chemokines by senescent cells, thereby helping to maintain a chronic inflammatory microenvironment [87].

In gingival and periodontal cell models, human gingival fibroblasts exposed to *P. gingivalis* or its lipopolysaccharide exhibit mitochondrial depolarization, elevated mtROS, and pronounced mtDNA release from mitochondria into the cytosol and extracellular space [17]. In osteocytes and osteoblast lineage cells of the alveolar bone, LPS stimulation or aging related stress can induce a decrease in mitochondrial membrane potential, a reduction in oxygen consumption rate, and increased ROS production, together with upregulation of p21 and a higher proportion of SA-β-gal positive cells. These senescent cells secrete high levels of IL-6, IL-1β, TNF-α, MMP-13, and RANKL, which promote osteoclast formation and accelerate bone loss [89, 90].

### Disrupted mitochondrial dynamics, impaired mitophagy, and inflammasome driven cell death

**Fig. 3:**
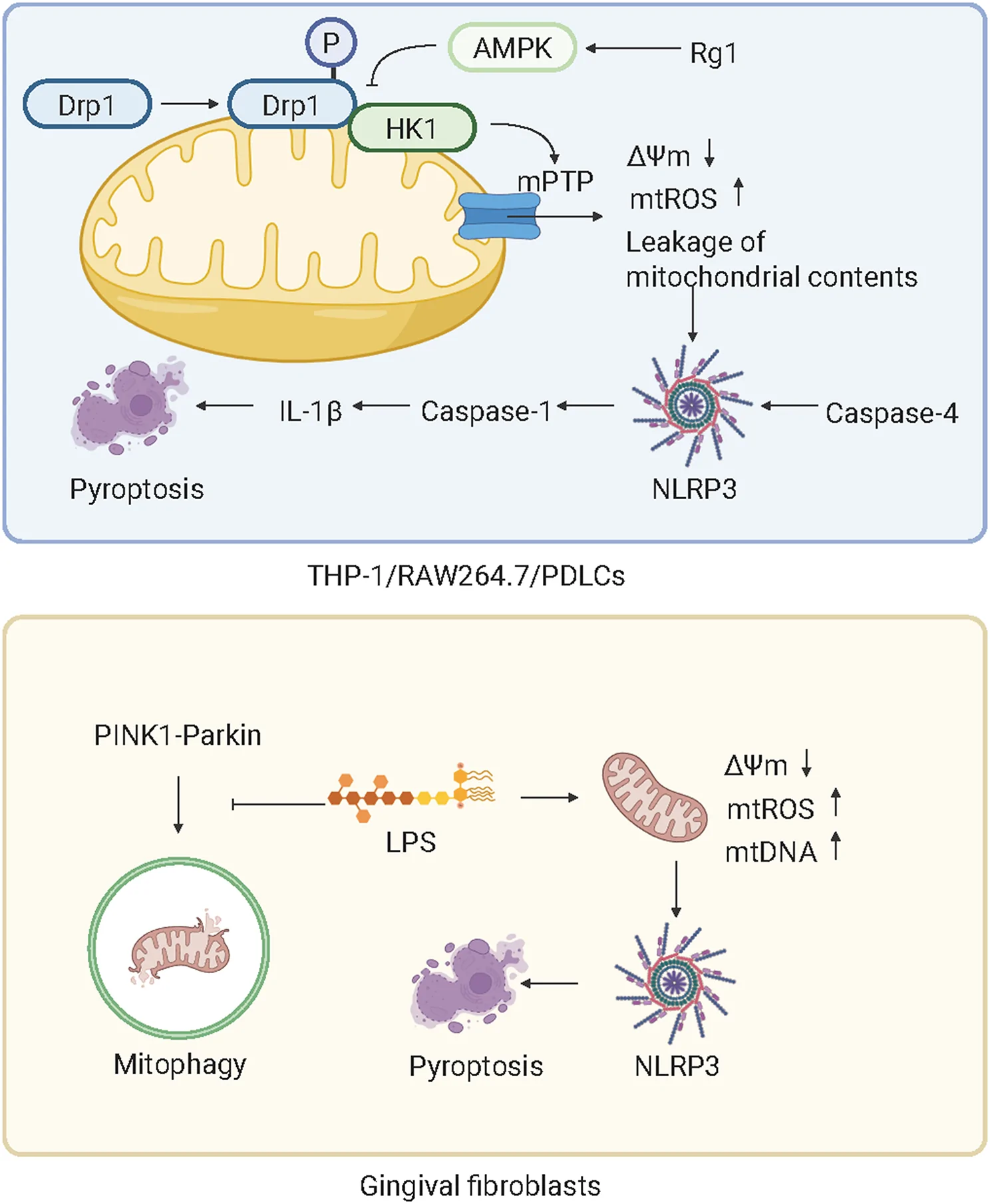
Drp1-mediated mitochondrial dynamics drive pyroptosis in periodontal inflammation. Under chronic periodontitis and aging-related conditions, sustained oxidative stress and metabolic disturbances promote Drp1-mediated excessive fission and insufficient fusion, causing the mitochondrial network to collapse into a fragmented state and ultimately triggering apoptosis. In a LPS-stimulated human periodontal ligament cell model, imbalanced mitochondrial dynamics drive inflammatory pyroptosis. Ginsenoside Rg1 activates AMPK signaling, promotes Drp1 phosphorylation at Ser637, and reduces its mitochondrial localization, thereby reversing LPS-induced mitochondrial hyperfission, mtROS bursts, and energy failure. This process simultaneously suppresses activation of the NLRP3/ASC/caspase-1/GSDMD pyroptotic pathway and the release of IL-1β and IL-18. In THP-1 and RAW264.7 macrophages stimulated with Pg or its LPS, Drp1 phosphorylation and its translocation to mitochondria are markedly enhanced, and Drp1 forms a complex with the outer mitochondrial membrane protein HK1, leading to aberrant activation of the mPTP. This results in loss of mitochondrial membrane potential, mtROS bursts, and leakage of mitochondrial contents, ultimately promoting NLRP3 inflammasome assembly, caspase-1 and GSDMD cleavage, and IL-1β release. When PINK1–Parkin-mediated mitophagy is inhibited by LPS in gingival fibroblasts, mitochondrial membrane potential decreases and mtROS and mtDNA progressively accumulate in the cytoplasm, accompanied by NLRP3 inflammasome activation and the occurrence of pyroptotic cell death. Drp1, dynamin-related protein 1; LPS, lipopolysaccharide; mtROS, mitochondria-derived reactive oxygen species; Rg1, ginsenoside Rg1; AMPK, AMP-activated protein kinase; NLRP3, NOD-like receptor family pyrin domain-containing 3; ASC, apoptosis-associated speck-like protein containing a CARD; caspase-1, cysteinyl aspartate-specific proteinase-1; GSDMD, gasdermin D; IL-1β, interleukin-1 beta; IL-18, interleukin-18; Pg, *Porphyromonas gingivalis*; THP-1, human monocytic leukemia cell line THP-1; RAW264.7, murine macrophage cell line RAW264.7; HK1, hexokinase 1; mPTP, mitochondrial permeability transition pore; PINK1, PTEN-induced kinase 1; Parkin, E3 ubiquitin–protein ligase Parkin; mtDNA, mitochondrial DNA

Within the mitochondrial quality control system, mitochondrial dynamics are a central mechanism for maintaining homeostasis in periodontal cells. Under physiological conditions, the fission protein Drp1 and the fusion proteins Mfn1, Mfn2, and OPA1 counterbalance each other to form a dynamic mitochondrial network. This network allows damaged mitochondrial components to be diluted through fusion and ultimately removed through mitophagy [26]. However, in the presence of chronic periodontitis and aging, persistent oxidative stress and metabolic disturbance disrupt this equilibrium. A typical feature is Drp1 mediated excessive fission with relatively insufficient fusion, which causes the interconnected tubular network to disintegrate into fragmented mitochondria (Fig. 3). This fragmentation is a key morphological hallmark of cellular senescence and is considered an early event in mitochondrial dysfunction in periodontitis [18]. In hydrogen peroxide induced stress models of human PDLCs, mitochondria show structural disruption, loss of membrane potential, impaired ATP synthesis, and massive accumulation of mtROS, ultimately leading to apoptosis. Antioxidant interventions can partially reverse these abnormalities, which suggests that oxidative stress promotes senescence and death of periodontal ligament cells by damaging mitochondrial structure and function and by shifting mitochondrial dynamics toward fission [27]. In lipopolysaccharide (LPS) models of human periodontal ligament cells, imbalance of mitochondrial dynamics is associated with energy metabolism defects and contributes to inflammatory pyroptosis. Studies have shown that the ginsenoside Rg1 activates AMPK signaling and promotes inhibitory phosphorylation of Drp1 at Ser637, which reduces Drp1 recruitment to mitochondria [91]. This mechanism reverses LPS induced excessive mitochondrial fission, mtROS bursts, and energy failure. At the same time, it suppresses activation of the NLRP3/ASC/caspase-1/GSDMD pyroptosis pathway and reduces the release of IL-1β and IL-18 [91]. These findings demonstrate that Drp1 dependent disruption of mitochondrial dynamics is a key driver that links mitochondrial dysfunction to pyroptotic cell death [91].

During chronic inflammatory or pathogen-induced disturbances in mitochondrial dynamics, periodontal macrophages may amplify local stimuli into sustained inflammatory responses. Jiang and colleagues [92]showed that in gingival tissues from periodontitis patients, as well as in THP-1 and RAW264.7 macrophages stimulated with *P. gingivalis* or its LPS, Drp1 phosphorylation and its translocation to mitochondria are markedly increased. These changes are associated with enhanced mitochondrial fission and opening of the mPTP. Drp1 forms a complex with the outer mitochondrial membrane protein hexokinase 1 (HK1), which results in abnormal activation of the mPTP, loss of mitochondrial membrane potential, mtROS bursts, and leakage of mitochondrial contents. These events promote NLRP3 inflammasome assembly, caspase-1 and GSDMD cleavage, and IL-1β release, whereas pharmacologic inhibition of Drp1 simultaneously reverses these changes [92]. In THP-1 models, *P. gingivalis* induced NLRP3 dependent IL-1β production relies on a caspase-4/NLRP3/caspase-1 cascade, which further underscores the central role of mitochondria centered NLRP3 signaling in macrophage driven inflammation in periodontal lesions [93]. When LPS suppresses PINK1/Parkin mediated mitophagy in gingival fibroblasts, mitochondrial membrane potential declines and mtROS and mtDNA progressively accumulate in the cytosol. These changes are accompanied by activation of the NLRP3 inflammasome and the onset of pyroptosis. In contrast, enhancement of mitophagy restores mitochondrial function, reduces mtROS and mtDNA burden, and downregulates NLRP3 as well as IL-1β and IL-18 expression and release, which collectively attenuate inflammatory cell death [94]. Infection of human endothelial cells with *P. gingivalis* likewise enhances Drp1 phosphorylation at Ser616 and its mitochondrial translocation [95]. This change converts a reticular mitochondrial network into a markedly fragmented morphology and is associated with elevated mtROS, reduced membrane potential, and lower ATP content. Treatment with the Drp1 inhibitor Mdivi-1 significantly reverses these alterations, suggesting that periodontal pathogens can induce disturbed mitochondrial dynamics and mitochondrial dysfunction in distant vascular endothelium, resembling stress-related changes observed in local periodontal tissues [95].

Taken together, these findings suggest that, in resident periodontal cells and endothelial cells exposed to inflammatory or pathogen-related stress, imbalance of mitochondrial dynamics and impaired mitophagy may promote sustained release of mtROS and oxidized mtDNA as damage-associated molecular patterns. These signals may contribute to persistent activation of the NLRP3 inflammasome, pyroptosis, and bone destruction [18, 26]. Within this overall framework, mitochondrial aging is not merely a decline in energy producing capacity. It also involves enhanced Drp1 mediated fission, reduced function of the fusion proteins Mfn1, Mfn2, and OPA1, and further suppression of mitophagy. These changes convert disturbances in mitochondrial dynamics into a continuous signaling axis characterized by persistent mtROS accumulation, pathological mPTP opening, and stepwise activation of the NLRP3 inflammasome [18, 26].

### Immunometabolic rewiring of macrophages and T cells in periodontal lesions

In the periodontal lesion microenvironment, immune cells undergo highly specific reprogramming of mitochondrial structure and metabolism, which reshapes both the intensity and duration of local inflammatory responses [96].

**Fig. 4:**
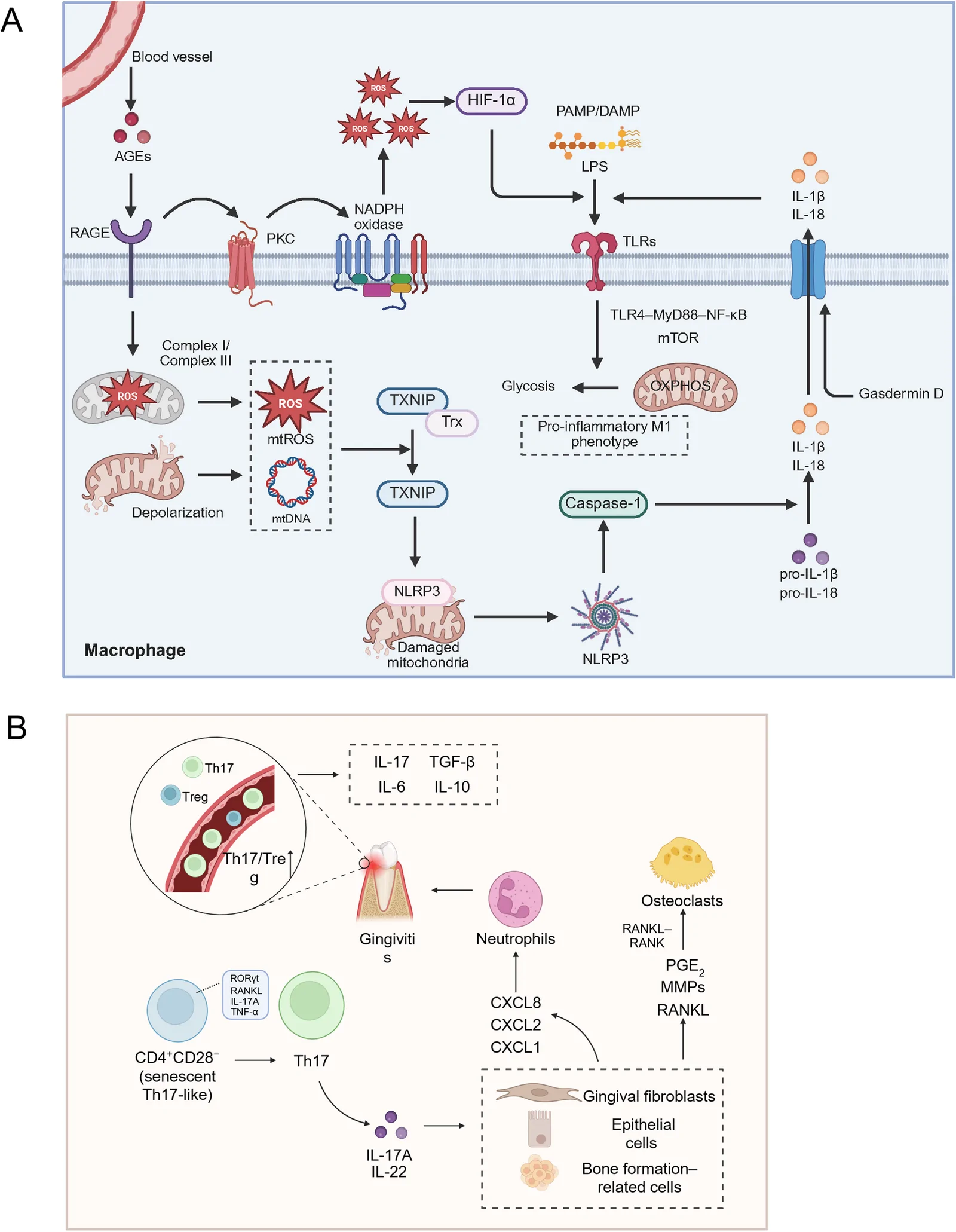
Integrated macrophage and Th17 pathways linking chronic inflammation to alveolar bone destruction. (**A**) Under chronic periodontitis and aging-related conditions, sustained oxidative stress and metabolic disturbance promote Drp1-mediated excessive fission and insufficient fusion, causing collapse of the mitochondrial network into a fragmented state and ultimately triggering apoptosis. In a LPS-stimulated human periodontal ligament cell model, imbalanced mitochondrial dynamics drive inflammatory pyroptosis. Advanced glycation end products derived from glucose bind to their receptor and, via the PKC pathway, activate NADPH oxidase while simultaneously damaging mitochondrial electron transport chain complexes I and III, leading to cooperative elevation of ROS originating from mitochondria and NADPH oxidase. These excessive ROS inhibit prolyl hydroxylase activity, render HIF-1α) resistant to degradation, and synergize with TLR4–MyD88–NF-κB and mTOR signaling triggered by lipopolysaccharides from periodontal pathogens, thereby driving macrophages to shift from an OXPHOS-dominated metabolic state to a highly glycolytic pro-inflammatory M1 phenotype and maintaining macrophages in the periodontitis microenvironment under a state of sustained inflammatory metabolic load. Mitochondria-derived ROS, together with concomitant mitochondrial depolarization and mtDNA release, induce TXNIP to dissociate from the thioredoxin complex and bind NLRP3, promoting the redistribution of NLRP3 from a diffuse cytosolic state to the vicinity of damaged mitochondria and thereby accelerating assembly of the inflammasome scaffold. The NLRP3 inflammasome recruits and activates caspase-1, mediates proteolytic maturation of pro-IL-1β and pro-IL-18, and induces Gasdermin D–mediated pore formation, leading macrophages and other immune cells to mount an amplified IL-1β and IL-18 secretory response to dental plaque–associated PAMP and DAMP signals, thereby enhancing destruction of periodontal hard and soft tissues. (**B**) In patients with gingivitis, the peripheral blood Th17/Treg ratio is increased, and metabolites are enriched in signaling networks related to Th17/Treg balance, including IL-17, TGF-β, IL-6, and IL-10. IL-17 A and IL-22 secreted by Th17 cells bind to receptors on gingival fibroblasts, epithelial cells, and osteogenic lineage cells, inducing these non-hematopoietic cells to upregulate RANKL, MMPs, and PGE2, and to continuously recruit neutrophils by promoting expression of chemokines such as CXCL1, CXCL2, and CXCL8. This enhances differentiation of osteoclast precursors into mature osteoclasts through the RANKL–RANK axis and amplifies neutrophil-dependent tissue-destructive responses. On this basis, aging-associated CD4⁺CD28⁻ T cells upregulate Th17-related factors such as RORγt, RANKL, IL-17 A, and TNF-α, as well as osteoclastogenic mediators including RANKL, thereby acquiring a Th17-like secretory profile characterized by high RANKL and IL-17 A, breaking the Th17/Treg balance and promoting osteoclastogenesis and alveolar bone resorption. Drp1, dynamin-related protein 1; LPS, lipopolysaccharide; PKC, protein kinase C; NADPH, nicotinamide adenine dinucleotide phosphate; ROS, reactive oxygen species; HIF-1α, hypoxia-inducible factor-1α; TLR4, Toll-like receptor 4; MyD88, myeloid differentiation primary response 88; NF-κB, nuclear factor kappa-B; mTOR, mechanistic target of rapamycin; OXPHOS, oxidative phosphorylation; M1, classically activated M1 macrophage; mtDNA, mitochondrial DNA; TXNIP, thioredoxin-interacting protein; NLRP3, NOD-like receptor family pyrin domain-containing 3; pro-IL-1β, pro–interleukin-1 beta; pro-IL-18, pro–interleukin-18; IL-1β, interleukin-1 beta; IL-18, interleukin-18; PAMP, pathogen-associated molecular pattern; DAMP, damage-associated molecular pattern; Th17, T helper 17 cell; Treg, regulatory T cell; IL-17, interleukin-17; TGF-β, transforming growth factor-β; IL-6, interleukin-6; IL-10, interleukin-10; IL-17 A, interleukin-17 A; IL-22, interleukin-22; RANKL, receptor activator of nuclear factor κB ligand; MMPs, matrix metalloproteinases; PGE2, prostaglandin E2; CXCL1, C-X-C motif chemokine ligand 1; CXCL2, C-X-C motif chemokine ligand 2; CXCL8, C-X-C motif chemokine ligand 8; RANK, receptor activator of nuclear factor κB; CD4, cluster of differentiation 4; CD28, cluster of differentiation 28; RORγt, RAR-related orphan receptor gamma t; TNF-α, tumor necrosis factor-α

In macrophages (Fig. 4A), classically activated inflammatory M1 cells typically show interruption of the tricarboxylic acid (TCA) cycle, reduced mitochondrial oxidative phosphorylation, and enhanced glucose dependent glycolysis. These changes are accompanied by elevated mitochondrial membrane potential and robust mtROS production, which together drive the generation of proinflammatory mediators such as IL-1β and TNF-α [97]. In contrast, tissue repair associated M2 macrophages depend more on an intact TCA cycle and fatty acid oxidation, maintain relatively high OXPHOS efficiency and low ROS levels, and thereby favor the production of anti-inflammatory and proresolving factors [96]. In gingival tissues from patients with periodontitis, macrophage mitochondria exhibit swelling, disrupted cristae, decreased membrane potential, and reduced oxygen consumption rates, together with a marked increase in mtROS. Intracellular mtDNA levels decrease, while extracellular cell free mtDNA increases, which indicates a shift from OXPHOS toward glycolysis dominated metabolism [26]. Under high glucose conditions, excessive glucose uptake and the interaction of advanced glycation end products with their receptors activate NADPH oxidase through protein kinase C dependent pathways and simultaneously damage mitochondrial electron transport chain complexes I and III. As a result, ROS derived from both mitochondria and NADPH oxidase increase cooperatively and disrupt intracellular redox balance [81]. These excessive ROS inhibit prolyl hydroxylase activity, impair degradation of hypoxia-inducible factor 1-α (HIF-1α), and synergize with TLR4–MyD88–NF-κB and mTOR signaling triggered by periodontal pathogen derived LPS. This synergy drives macrophages from an OXPHOS based metabolic state into a highly glycolytic proinflammatory M1 phenotype and maintains macrophages in periodontitis lesions under a sustained inflammatory metabolic burden [81]. Mitochondria derived ROS, together with mitochondrial depolarization and mtDNA release, can induce dissociation of TXNIP from the thioredoxin complex and promote its binding to NLRP3. This process facilitates recruitment of NLRP3 from a diffuse cytosolic distribution to damaged mitochondria and accelerates assembly of the inflammasome scaffold [26]. On this basis, the NLRP3 inflammasome recruits and activates caspase-1, which cleaves pro-IL-1β and pro-IL-18 into their mature forms and induces Gasdermin D mediated pore formation. These events amplify IL-1β and IL-18 secretion in response to plaque associated PAMP and DAMP signals in macrophages and other immune cells and thereby enhance destructive responses against periodontal hard and soft tissues [26].

In CD4⁺ T cells, Th17 cells depend heavily on aerobic glycolysis and glutaminolysis, whereas regulatory T cells (Tregs) rely more on fatty acid oxidation and intact mitochondrial OXPHOS to maintain their suppressive function [98]. Mitochondrial dysfunction or elevated mtROS weaken the ability of Tregs to use oxidative phosphorylation and NAD⁺ dependent deacetylation pathways and, at the same time, enhance HIF-1α and mTOR signaling. This combination promotes Th17 differentiation and IL-17 production and disrupts the Th17/Treg balance [98]. Clinical studies have found an increased Th17/Treg ratio in peripheral blood from patients with gingivitis (Fig. 4B), together with significant changes in plasma metabolites related to glycolysis, amino acid metabolism, and fatty acid metabolism. Pathway and IPA analyses show that these metabolic changes are enriched in IL-17, TGF-β, IL-6, IL-10, and other signaling networks related to Th17/Treg regulation, suggesting a close link between immunometabolic imbalance and Th17/Treg disequilibrium [99]. In diseased gingiva from periodontitis patients, Th17 cells secrete IL-17 A and IL-22, which bind to receptors on gingival fibroblasts, epithelial cells, and osteogenic cells. These interactions induce upregulation of RANKL, MMPs, and PGE2 in nonhematopoietic cells and promote expression of chemokines such as CXCL1, CXCL2, and CXCL8, which continuously recruit neutrophils. In this way, Th17 responses simultaneously enhance the differentiation of osteoclast precursors into mature osteoclasts through the RANKL–RANK axis and amplify neutrophil mediated tissue damage. This pathway constitutes a key inflammatory link between Th17 metabolic skewing and alveolar bone resorption [100]. In addition, aging related CD4⁺CD28⁻ T cells undergo ATM mediated DNA damage responses and sustained p38 MAPK activation, accompanied by suppressed autophagy, accumulation of dysfunctional mitochondria, and increased ROS. At the transcriptional level, these cells upregulate Th17 related factors such as RORγt, RANKL, IL-17 A, and TNF-α and increase expression of osteoclastogenic molecules including RANKL. As a result, they acquire a Th17-like secretory profile enriched in RANKL and IL-17 A, which both disrupts the Th17/Treg balance and promotes osteoclast formation and alveolar bone resorption [101].

### Extracellular vesicle mediated dissemination of mitochondrial injury and protective signals

In periodontitis, mitochondrial damage is not confined to individual cells. It can also propagate between cells via extracellular vesicles. Through this route, mitochondrial injury reshapes the periodontal stem cell niche and drives the imbalance between inflammation and bone resorption toward a detrimental direction [26]. Therefore, these vesicles are not merely conventional paracrine mediators but may also represent an intercellular communication route that emerges when mitochondrial quality control is impaired or overloaded. Consistent with this view, recent aging-related reviews have proposed that mitoEVs and MDVs can transport mitochondria-derived cargo, including mitochondrial proteins, mtDNA, and other mitochondrial components, thereby participating in mitochondrial quality control, inflammatory signaling, and the progression of age-related diseases [102, 103]. In the inflammatory microenvironment of periodontitis, extracellular vesicles may therefore serve as a key link connecting local inflammatory stress, intercellular propagation of mitochondrial injury, and the formation of an aging-like periodontal microenvironment.

In an experimental periodontitis model established in C57BL/6 mice by ligation of the maxillary molars combined with topical application of *P. gingivalis*, macrophages in the lesion area first exhibit loss of mitochondrial membrane potential and upregulation of inflammatory cytokines. These macrophages then package large amounts of damaged mitochondria into extracellular vesicles expressing CD29 and CD63, forming mitochondria-rich extracellular vesicles. In both in vitro coculture and in vivo tail vein injection experiments, these vesicles are efficiently taken up by bone marrow mesenchymal stem cells (BMSCs) [104]. In an in vitro coculture system, BMSCs that internalize mitochondria-rich vesicles from inflammatory macrophages undergo a transition from a relatively intact reticular mitochondrial network to a distinctly fragmented morphology. This is accompanied by disruption of the inner membrane protein OPA1, reduced mitochondrial membrane potential, and decreased ATP production. Functionally, alkaline phosphatase staining and mineralized nodule formation are reduced. The expression of osteogenic genes, including Runx2 and OCN, is also downregulated. This indicates that mitochondrial damage in inflammatory cells during periodontitis can be transferred to osteogenesis-related stem cells via vesicles, causing aging-like functional decline in recipient cells, including disrupted mitochondrial dynamics, reduced energy production, and impaired regenerative capacity [104]. Proteomic and functional analyses further show that after inflammatory macrophage-derived vesicles enter BMSCs, expression of the lipid carrier protein LCN2 is markedly upregulated. Elevated LCN2 activates the mitochondrial inner membrane protease OMA1. This disrupts the OMA1–OPA1 axis, causing abnormal OPA1 cleavage and impaired mitochondrial cristae remodeling. LCN2 therefore emerges as a key molecular node that links mitochondrial structural disruption to impaired osteogenesis [104]. In the same mouse periodontitis model, tail vein injection of mitochondria-rich extracellular vesicles from inflammatory macrophages further increases the distance from the cementoenamel junction to the alveolar bone crest and decreases alveolar bone volume fraction, indicating that these vesicles can amplify inflammation-related bone resorption in vivo [104]. Under continuous administration of the same vesicles, genetic knockout or pharmacologic inhibition of LCN2 partially restores mitochondrial morphology and osteogenic differentiation capacity in BMSCs and significantly attenuates alveolar bone loss [104]. In sharp contrast to this detrimental mitochondrial transfer, exosomes derived from PDLSCs can, under specific conditions, deliver antioxidant and anti-inflammatory signals to surrounding cells through their nucleic acid cargo. In a high glucose-induced senescence model of human PDLSCs, treatment with 25 mmol/L glucose increases SA-β-gal activity, upregulates p16, p21, and γH2AX, and elevates intracellular ROS levels. Exosomes from healthy PDLSCs reverse these senescence-associated changes, restore cell proliferation, and rescue the expression of osteogenic differentiation-related genes [105]. Mechanistic studies reveal that these exosomes are enriched in miR-141-3p, which can be internalized by recipient cells. miR-141-3p targets and suppresses KEAP1, thereby activating the NRF2-mediated antioxidant pathway, upregulating downstream genes such as HO-1, alleviating high glucose-induced oxidative stress, and ultimately enhancing the resistance of PDLSCs to mitochondrial damage and inflammatory stimuli [105]. Moreover, in peripheral blood and local lesion tissues from patients with chronic periodontitis, PDLSC-derived exosomes carrying miR-155-5p can target and inhibit SIRT1, modulate the Th17/Treg immune balance, and reduce levels of inflammatory cytokines including IL-17 and IL-6, thereby improving the local immune microenvironment in periodontal tissues [45].

Overall, extracellular vesicles may serve as intercellular carriers of mitochondrial aging-like signals in periodontitis. Mitochondria-enriched vesicles derived from inflammatory macrophages can transfer damaged mitochondria, disrupt mitochondrial dynamics in BMSCs, and promote osteogenic decline. Conversely, exosomes derived from healthy PDLSCs or young dental cells may alleviate stress-induced aging-like phenotypes through antioxidant signaling or remodeling of mitochondrial dynamics.

### PDLSC mitochondrial aging, mitophagy, and age dependent bone remodeling and tooth movement

Animal and in vitro studies indicate that mitochondrial responses of PDLSCs to compressive and tensile forces decline systematically with aging. In a model comparing 6-week-old (adolescent) and 12-month-old (adult) rats, application of identical nickel-titanium spring traction results in significantly greater tooth movement in the adolescent group. At the pressure side of the alveolar bone, expression levels of the mitophagy-related proteins PINK1 and Parkin, as well as the numbers of TRAP-positive osteoclasts and osteocalcin-positive osteoblasts, increase more markedly in adolescent rats than in adults. These observations suggest that force-induced mitophagy and the coupled bone remodeling response are attenuated in older individuals [21]. In the same study, PDLSCs were isolated from adolescent and adult human donors. After exposure to constant compressive force, both groups showed increased PINK1/Parkin-dependent mitophagy. However, PDLSCs from adult donors exhibited a lower level of mitophagy, along with reduced mitochondrial membrane potential, decreased ATP production, and increased ROS accumulation. In addition, the compressive force-induced increase in the RANKL/OPG ratio was significantly blunted in the adult group [21]. To validate the underlying mechanism, small interfering RNA was used to knock down PINK1 or Parkin in young PDLSCs, which successfully reproduced the adult phenotype characterized by mitochondrial dysfunction and a reduced RANKL/OPG ratio. These results demonstrate that insufficient mitophagy weakens the ability of pressure-side PDLSCs to deliver pro-osteoclastogenic signals and directly slows orthodontic tooth movement (OTM) [21]. At the animal level, injection of the mitophagy inducer urolithin A enhances alveolar bone resorption and formation and significantly accelerates tooth movement in aged rats, whereas administration of the mitochondrial fission inhibitor Mdivi-1 further delays tooth movement and suppresses bone remodeling. Together, these cellular and in vivo findings establish mitophagy as a critical determinant of OTM speed [21]. Mitochondrial aging of PDLSCs is increasingly recognized as a central hub linking age-related weakening of bone responsiveness to increased susceptibility to periodontitis. In a rat model of age-related alveolar bone defects and a parallel in vitro model of passaging-induced PDLSC aging, senescent PDLSCs display persistently low mitophagy, decreased mitochondrial membrane potential, reduced respiratory chain activity, and ROS accumulation, along with markedly impaired osteogenic differentiation. Consistently, in aged mice, alveolar bone defects show insufficient new bone formation accompanied by attenuated expression of mitophagy markers [106]. Enhancing Parkin acetylation-related mitophagy, for example by inhibiting HDAC2-mediated deacetylation of Parkin, restores mitochondrial quality control in aging PDLSCs, improves mitochondrial function, and significantly enhances osteogenic differentiation in vitro. In aged mouse models, this intervention also promotes regeneration of alveolar bone defects, indicating that age-related decline in bone remodeling capacity is largely attributable to impaired Parkin-dependent mitophagy [106]. In diabetic periodontitis models, human PDLSCs exposed to a high glucose inflammatory environment develop pronounced cellular senescence, mitochondrial dysfunction, and extensive mtDNA leakage, which leads to sustained activation of the cGAS-STING pathway, exhaustion of the stem cell pool, and exacerbation of periodontal bone destruction [107]. Local injection of the small molecule spermidine promotes mitophagy, facilitates clearance of damaged mitochondria, reduces mtDNA release and STING phosphorylation, restores mitochondrial function in PDLSCs, mitigates cellular senescence, and improves periodontal bone mass. Notably, these beneficial effects are abolished when mitophagy is experimentally blocked, confirming that spermidine acts through a mitophagy-dependent mechanism [107].

Integrating these findings, it can be inferred that in younger individuals, appropriate compressive forces induce PINK1/Parkin-mediated mitophagy in PDLSCs, which promptly clears mitochondria damaged by mechanical stress. This allows PDLSCs to adequately upregulate RANKL and moderately downregulate OPG, thereby achieving rapid and balanced bone remodeling and tooth movement [21, 108]. In contrast, in adults or in individuals with chronic inflammation in whom PDLSCs have undergone mitochondrial aging, both basal and mechanically induced mitophagy are reduced. Damaged mitochondria are cleared inefficiently, the capacity to increase the RANKL/OPG ratio is limited, and the clinical phenotype manifests as slow orthodontic tooth movement and unstable bone mass.

## Mitochondria targeted therapeutic approaches for periodontitis

Given that mitochondrial dysfunction may contribute to persistent inflammation and tissue destruction in periodontitis, an increasing number of studies began to explore strategies that restore mitochondrial homeostasis in order to normalize energy metabolism and redox balance in the periodontal microenvironment. These approaches include mitochondria-targeted antioxidants such as MitoQ, resveratrol, and coenzyme Q10. More recently, mitochondrial transplantation and engineered exosomes have also been developed to target mitochondria and reprogram cellular metabolism within periodontal lesions. Current mitochondria based therapeutic strategies for periodontitis are summarized in Table 1.

**Table 1:** Mitochondria-targeted therapeutic strategies for periodontitis

Intervention	Model	Mechanism/Effects	References
MitoQ@PssL (MitoQ-loaded ROS-responsive nanoparticles)	LPS-stimulated human periodontal ligament stem cells (hPDLSCs); SD/Wistar rats with ligatures around maxillary molars plus local *P. gingivalis* LPS injection to induce experimental periodontitis	Mitochondria-targeted ROS scavenging, activation of PINK1–Parkin mitophagy, promotes osteogenesis, reduces alveolar bone loss	[69]
Resveratrol (administered in drinking water)	Wistar rat experimental periodontitis model induced by ligature around maxillary molars (long-term resveratrol in drinking water)	Activates Sirt1/AMPK and Nrf2 pathways, decreases local and systemic oxidative stress, inhibits alveolar bone resorption	[109]
Resveratrol (gavage, 50 mg/kg·day for 8 weeks)	Experimental periodontitis in Wistar rats induced by steel-wire ligature (resveratrol gavage 50 mg/kg·day for 8 weeks)	Improves renal mitochondrial structure and membrane potential, restores ATP, reduces oxidative stress, and simultaneously improves periodontal clinical parameters	[110]
Resveratrol solid lipid nanoparticle–hyaluronic acid hydrogel	THP-1 human monocytic cell–derived M1/M2 macrophages and RANKL-induced osteoclast-like cells; Osteogenic-induced human dental pulp stem cells (hDPSCs); High glucose (25 mmol/L) and *P. gingivalis* LPS in a diabetes-related periodontitis in vitro simulation model	Reduces ROS, restores mitochondrial membrane potential, promotes osteogenic differentiation of osteoblast-like cells, inhibits formation of osteoclast-like cells	[111]
Coenzyme Q10 gel (local intra-pocket administration)	Adult smokers with chronic periodontitis, randomized controlled clinical trial; both groups received scaling and root planing, test group additionally received intra-pocket coenzyme Q10 gel	Enhances mitochondrial electron transport and ATP synthesis, decreases local oxidative damage, improves periodontal inflammation and attachment loss	[114]
M2 macrophage–derived mitochondrial transplantation (M2-MT)	*P. gingivalis* LPS-stimulated hPDLSCs co-cultured with mitochondria from M2 macrophages; SD rats with experimental periodontitis induced by 4 − 0 silk ligature around maxillary second molars for 21 days plus local injection of M2-MT	Restores OXPHOS, enhances ATP production, attenuates ROS, activates p38–MAPK, suppresses HIF-1α, and promotes osteogenesis and periodontal bone regeneration.	[116]
FoxO1-overexpressing hPDLSC-derived small extracellular vesicles (FoxO1 sEV)	hPDLSCs and THP-1 cells treated with LPS/H₂O₂ plus different sEV preparations; C57BL/6 mouse experimental periodontitis model induced by ligature around maxillary second molars with local injection of PDLSC-derived FoxO1 sEV	Reduces ROS, restores ATP and mitochondrial membrane potential, enhances mitochondrial respiration, promotes osteogenic differentiation and induces M2 macrophage polarization	[117]

### Pharmacologic modulation of mitochondrial homeostasis: MitoQ, resveratrol, and coenzyme Q10

MitoQ is a mitochondria-targeted antioxidant in which a ubiquinone moiety is covalently linked to a positively charged triphenylphosphonium group. Driven by the mitochondrial membrane potential, MitoQ selectively accumulates within mitochondria and is widely considered capable of scavenging mitochondria-derived ROS and preserving mitochondrial function [69]. In a study reported by Li and colleagues, the authors developed a controlled release nanoparticle system loading MitoQ (MitoQ@PssL) and applied it to human PDLSCs exposed to lipopolysaccharide in order to mimic the oxidative stress environment of periodontitis. MitoQ@PssL treatment improved cell viability and osteogenic phenotypes and reduced intracellular oxidative stress, indicating that low dose mitochondria-targeted delivery is sufficient to confer antioxidant and cytoprotective effects [69]. In the same study, MitoQ@PssL was found to activate PINK1/Parkin mediated mitophagy and to promote clearance of damaged mitochondria, which is beneficial for maintaining intracellular homeostasis [69]. In animal models of periodontitis, MitoQ@PssL therapy attenuates alveolar bone loss and inflammatory cell infiltration and reduces markers of oxidative stress. These observations suggest that its in vivo effects are mainly achieved by suppressing excessive oxidative stress and modulating mitochondrial quality control, thereby limiting periodontal bone destruction [69]. Taken together, MitoQ represents a local delivery strategy that directly targets mitochondria. It can provide sustained antioxidant and mitochondria-protective effects at relatively low doses and has potential as a candidate molecule for local controlled release formulations in periodontal therapy [69].

Resveratrol is a prototypical polyphenolic compound with strong antioxidant and anti-inflammatory activities. It can also activate SIRT1–AMPK and NRF2 signaling pathways and thereby regulate mitochondrial biogenesis and antioxidant defense systems. In periodontitis related models, resveratrol has been shown to ameliorate both local and systemic oxidative stress and is closely associated with preservation of mitochondrial function [109]. In one study, Wistar rats were subjected to wire ligature-induced periodontitis and received resveratrol by oral gavage at a daily dose of approximately 50 mg/kg for 8 weeks. In untreated periodontitis animals, renal mitochondria exhibited marked structural damage, including disorganized cristae, loss of membrane potential, accumulation of mitochondrial ROS, and reduced ATP content, together with downregulation of SIRT1 and PGC-1α. In contrast, resveratrol-treated rats showed clear improvement in these mitochondrial injury parameters, restoration of mitochondrial membrane potential and ATP production, and reduced oxidative stress in renal tissue. Periodontal clinical outcomes, such as probing depth, gingival bleeding, and alveolar bone height, were also improved compared with untreated animals. These findings suggest that periodontitis induces systemic oxidative stress that damages renal mitochondria and that resveratrol can partially improve both periodontal status and mitochondrial function in distant organs [110]. In addition, Conte and colleagues developed a resveratrol-loaded solid lipid nanoparticle system incorporated into a hyaluronic acid hydrogel and applied it to immune and bone cells related to periodontal tissues under conditions of high glucose and inflammation. This system reduced intracellular ROS, restored mitochondrial membrane potential, promoted osteoblast-like cell differentiation, and inhibited RANKL-induced osteoclast formation. In models of diabetes-associated periodontitis, this hydrogel shows promise as a local multitarget strategy that combines immunomodulatory, antioxidant, and pro-osteogenic effects [111].

Coenzyme Q10 (CoQ10) is a key cofactor in the mitochondrial electron transport chain that transfers electrons between respiratory complexes and, in its reduced form, has strong antioxidant capacity. It can directly scavenge free radicals generated during lipid and protein oxidation and is therefore regarded as a typical mitochondria-directed antioxidant molecule [112]. Clinically, Rasoolzadeh and colleagues performed a systematic review and meta-analysis of 11 trials investigating local CoQ10 gel as an adjunct to scaling and root planing. Compared with mechanical debridement alone, adjunctive CoQ10 gel provided additional reductions in plaque index, gingival bleeding index, probing depth, and clinical attachment loss, with standardized mean differences generally in the range of several tenths to around one [113]. Subgroup analyses suggested that intrapocket application yielded more pronounced benefits than simple surface application, indicating that achieving higher local CoQ10 concentrations within periodontal pockets favors its antioxidant action [113]. Raut and colleagues conducted a randomized controlled trial in smokers with chronic periodontitis. Forty participants received scaling and root planing; the test group additionally received local CoQ10 gel, whereas the control group received mechanical treatment alone. At 1 and 3 months of follow-up, plaque index, gingival bleeding index, probing depth, and clinical attachment level improved significantly from baseline in the test group and showed an overall better trend than in the control group [114]. Mechanistically, basic and review studies generally support that CoQ10 enhances mitochondrial electron transport and ATP synthesis, reduces the generation of superoxide in the intermembrane space, and stabilizes mitochondrial membrane potential. These effects decrease oxidative damage in periodontal tissues and help maintain the survival and matrix-synthesizing ability of fibroblasts and osteoblasts, thereby indirectly inhibiting osteoclast activation and alveolar bone resorption. This mitochondria-centered protective mechanism provides a strong biological rationale for using CoQ10 as an adjunctive agent in periodontitis therapy [112, 113].

### Organelle based and exosome-based strategies: mitochondrial transplantation and engineered PDLSC derived vesicles

Mitochondrial transplantation is an emerging organelle based therapeutic strategy. Its core concept is to isolate functional mitochondria from metabolically healthy donor cells and transfer them into target cells with mitochondrial dysfunction in order to rapidly restore their energy metabolism [115]. Ma and colleagues were the first to systematically evaluate the effects of mitochondrial transplantation from M2 macrophages on human PDLSC function and periodontal bone regeneration in a periodontitis model. In vitro, the authors created an inflammatory microenvironment by stimulating PDLSCs with *P. gingivalis* LPS and then cocultured these cells with intact mitochondria isolated from M2 macrophages. Compared with cells exposed to LPS alone, PDLSCs receiving mitochondrial transplantation showed significantly increased expression of osteogenic genes and proteins, including alkaline phosphatase (ALP), RUNX2, and OCN, along with an increased number of mineralized nodules. At the metabolic level, intracellular ROS decreased, mitochondrial membrane potential increased, ATP content rose, and extracellular acidification rate declined, indicating that mitochondrial transplantation restored oxidative phosphorylation and suppressed excessive glycolysis, thereby reconstructing metabolic homeostasis [116]. Transcriptomic and protein expression analyses further suggested that this process depends primarily on activation of the p38 MAPK signaling pathway. A specific p38 inhibitor markedly attenuated the promotive effects of mitochondrial transplantation on osteogenic differentiation and ALP activity. In addition, the study observed that HIF-1α, which is highly expressed under inflammatory conditions, was significantly downregulated after mitochondrial transplantation. This finding indicates that the strategy can relieve the inhibitory effects of the inflammatory microenvironment on mitochondrial function by suppressing HIF-1α–related metabolic stress pathways [116].

Niu and colleagues used genetic engineering to overexpress the transcription factor forkhead box protein O1 (FoxO1) in human PDLSCs and then isolated three types of small extracellular vesicles (sEVs): Nor sEV from unmodified PDLSCs, NC sEV from vector-transfected cells, and FoxO1 sEV from FoxO1-overexpressing cells. These exosomes were applied at a protein concentration of 50 µg/mL to LPS-prestimulated hPDLSCs and THP-1 cells for 48 h to systematically evaluate their effects on mitochondrial structure and function [117]. In hPDLSCs, stimulation with LPS or H₂O₂ markedly increased intracellular ROS levels, reduced ATP production, and induced structural mitochondrial damage, including cristae disruption and swelling. In contrast, FoxO1 sEV treatment significantly attenuated ROS signals, restored ATP levels, and increased the proportion of morphologically intact mitochondria on transmission electron microscopy [117]. JC-1 staining showed that LPS caused mitochondrial membrane depolarization, whereas all sEV treatments reversed this effect, with FoxO1 sEV exerting the strongest restorative impact on membrane potential. Seahorse metabolic analysis further demonstrated that FoxO1 sEV significantly enhanced basal respiration, maximal respiration, ATP-linked oxygen consumption, and spare respiratory capacity in an inflammatory environment, indicating a global improvement in mitochondrial respiratory chain function [117]. This study provides direct evidence that engineered PDLSC-derived exosomes can deliver FoxO1 to periodontal cells and effectively correct mitochondrial structural abnormalities and restore respiratory function at the cellular level.

## Conclusions and future perspectives

This review explores the relationship between mitochondrial function and the onset and progression of periodontitis. By integrating tissue- and cell-level evidence, this review highlights the roles of ROS accumulation, disturbed energy metabolism, mitochondrial genome damage, and altered mitochondrial dynamics. It further links these changes to inflammatory amplification, imbalance of bone remodeling, and cellular senescence. Existing data further indicate that extracellular vesicle–mediated transfer of mitochondrial components serves as a hub for propagating damage signals between cells and reshaping the periodontal stem cell niche, and that this process also influences orthodontic tooth movement and bone repair. Together, these findings support the concept that mitochondrial functional decline is a core bridge connecting cellular senescence with a senescence-associated secretory phenotype. Finally, this review summarizes mitochondria-targeted therapeutic strategies and proposes an integrated framework in which mitochondrial imbalance is considered an important component of periodontal inflammation, tissue degeneration, and therapeutic response.

Current evidence suggests that mitochondrial dysfunction may represent a key node linking plaque-derived stimuli, host inflammatory responses, age related senescence, and multisystem comorbidities. However, several important questions remain unresolved. Much of the current evidence comes from cross-sectional human samples, reductionist in vitro systems, or individual animal models, which limits our ability to define whether mitochondrial alterations initiate periodontal inflammation, amplify ongoing tissue damage, or arise as secondary consequences of the inflammatory milieu. A related challenge is conceptual: mitochondrial dysfunction induced by bacterial products, inflammatory cytokines, hyperglycaemia, or oxidative stress should not be directly equated with chronological mitochondrial ageing, even though these processes may converge within chronically inflamed periodontal tissues. The temporal and spatial dynamics of mitochondrial states across distinct periodontal cell populations also remain poorly defined. Future studies combining single-cell and spatial omics with high-resolution mitochondrial imaging will be needed to determine how mitochondrial dysfunction evolves across different cell types, disease stages, ages, and metabolic backgrounds. In parallel, mitochondrial-related biomarkers in saliva or gingival crevicular fluid require prospective validation before they can be used for risk stratification, disease monitoring, or therapeutic decision-making. Finally, although mitochondria-targeted compounds, mitochondrial transplantation, and engineered exosomes offer promising therapeutic directions, most remain preclinical or adjunctive approaches. Their optimal timing, dosage, delivery route, durability, long-term safety, and immunological consequences will need to be carefully evaluated before clinical translation.

## Data Availability

Not applicable.
